# Proportional Microvalve Using a Unimorph Piezoelectric Microactuator

**DOI:** 10.3390/mi11020130

**Published:** 2020-01-24

**Authors:** Arun Gunda, Gürhan Özkayar, Marcel Tichem, Murali Krishna Ghatkesar

**Affiliations:** Department of Precision and Microsystems Engineering, Delft University of Technology, 2600 AA Delft, The Netherlands; arun.gunda62637@gmail.com (A.G.); g.ozkayar@tudelft.nl (G.Ö.); m.tichem@tudelft.nl (M.T.)

**Keywords:** microvalve, microactuator, piezoelectric, unimorph, stereolithography, 3D-printing

## Abstract

Microvalves are important flow-control devices in many standalone and integrated microfluidic applications. Polydimethylsiloxane (PDMS)-based pneumatic microvalves are commonly used but they generally require large peripheral connections that decrease portability. There are many alternatives found in the literature that use Si-based microvalves, but variants that can throttle even moderate pressures (1 bar) tend to be bulky (cm-range) or consume high power. This paper details the development of a low-power, normally-open piezoelectric microvalve to control flows with a maximum driving pressure of 1 bar, but also retain a small effective form-factor of 5 mm × 5 mm × 1.8 mm. A novel combination of rapid prototyping methods like stereolithography and laser-cutting have been used to realize this device. The maximum displacement of the fabricated piezoelectric microactuator was measured to be 8.5 μm at 150 V. The fabricated microvalve has a flow range of 0–90 μL min^−1^ at 1 bar inlet pressure. When fully closed, a leakage of 0.8% open-flow was observed with a power-consumption of 37.5 μW. A flow resolution of 0.2 μL min^−1^—De-ionized (DI) water was measured at 0.5 bar pressure.

## 1. Introduction

The need for techniques that more efficiently utilize chemical and biological reagents in chemical analysis systems led to the introduction of micro-total analysis systems (μTAS) [1]. The versatility of miniaturizing fluidics was realized and subsequently utilized in applications like drug-delivery [2], micro- and nano-spacecraft thermal cooling and propulsion systems [3], and lab-on-a-chip devices [4]. An essential component in integrated microfluidic devices is the microvalve. In conjunction with a pressure source, it is used to control, direct, or regulate the pressure or flow rate of media within these microfluidic circuits. Valves that operate in the μm- to cm-length scales are generally classified as microvalves and they are usually fabricated using microfabrication techniques like (soft-)lithography and etching. Typically, microvalves contain a flow-channel that is obstructed by an active/passive element. Active microvalves, in contrast to their passive counterparts, have a controllable element within the device. These valves are usually classified by the working principle of the active element. One of the first reported microvalves used an electromagnetic solenoid actuator to control the separation between a valve-membrane and inlet-orifice [5]. A valve membrane or valve plate has since been one of the most used valving mechanisms to control fluid flow, with large variations in the actuation scheme. Principles like electrostatic actuation [6], piezoelectric actuation [7], pneumatic actuation [8], and thermopneumatic actuation [9] have been used with varying degrees of success.

In this work, a proportional microvalve was designed, fabricated and characterized. It is based on a piezoelectric actuation method that uses a commercially available piezoelectric plate as the active element. As the active material functions without any further processing, all the advantages of piezoelectric actuation were retained. The valve deflection was controlled by applying a desired voltage across the piezoelectric material, resulting in proportional control of the fluid flow rate. The following sections detail the design, fabrication and characterization of the microvalve.

## 2. Materials and Methods

**Figure 1:**
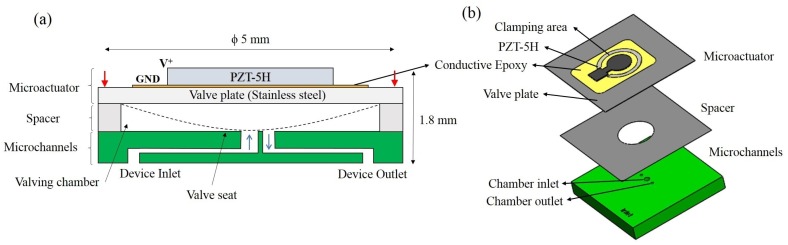
(**a**) Schematic of the microvalve: It consists of a circular piezoelectric unimorph microactuator, a spacer, and 3D-printed microchannels. The microactuator is placed on top of the microchannels with an intermediate spacer and the entire assembly is clamped. Red arrows indicate the direction of the clamping force. Black dashed lines indicate the membrane deformation when actuated. (**b**) Exploded 3D view of the microvalve.

A suspended piezoelectric microvalve with a small footprint of 5 mm × 5 mm × 1.8 mm was developed in this work. It can withstand a pressure of 1 bar and is compatible with both liquids and gases. A lateral dimensional constraint of 5 mm × 5 mm was assumed initially using prior literature as a reference to obtain a small device footprint [10]. As shown in Figure 1a, the piezoelectric actuator modulates the flow rate in the microvalve by closing the inlet orifice upon actuation. The fabrication approach that was used is a hybrid-integration method, using a combination of 3D-printing, laser-machining and epoxy-bonding to achieve the desired device. No microvalve was found in the literature that uses this combination of rapid prototyping methods and piezoelectric actuation mechanism.

As shown in Figure 1, a thin Lead Zirconate Titante (PZT) plate was laser-cut to the desired shape and was glued with conductive epoxy to a stainless steel (SS) foil to form a unimorph piezoelectric microactuator (UPM). A detailed study of the UPM is given in Section 2.1. A similar actuation scheme was used by Sobocinski et al in a microvalve manufactured using low-temperature co-fired ceramics (LTCC) [11]. In our device, the microchannels were printed using stereolithography, a 3D-printing technique that uses ultraviolet (UV)-light to selectively polymerize a UV-sensitive resin layer-by-layer. The spacer in the middle defined the initial separation between the actuator membrane and valve seat. A detailed explanation of the microchannels and spacer is given in Section 2.2. The entire assembly was clamped for the microvalve to function as designed. A clamping valve holder was 3D-printed with fluidic and electrical interfaces to an external test set-up. These are detailed in Section 2.3.

### 2.1. Microactuator

A unimorph piezoelectric microactuator (UPM) has been designed and fabricated. A unimorph refers to an actuator that has an active layer that is bonded to a passive substrate. Deformation of the active layer due to electrical stimulus causes a bending stress in the passive substrate. In this work, the active piezoelectric layer was Lead Zirconate Titanate (PZT-5H, Piezo Systems T105-H4E-602 [12]) and the passive layer was a stainless steel plate (Jeveka H+S 0.05 mm flat sheet). The PZT-5H plate was provided with vacuum-deposited nickel electrodes on both sides. A micro laser-etching machine (Optec, Frameries, Belgium) was used to laser-cut the PZT.

**Figure A1:**
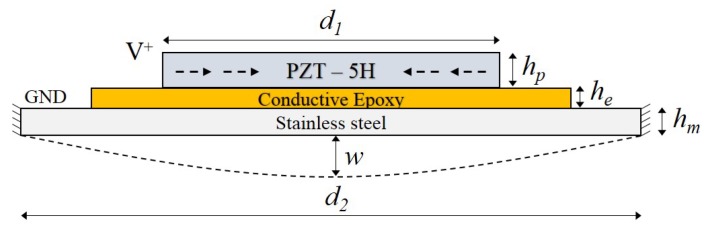
Schematic of the piezoelectric unimorph microactuator: Design parameters are the PZT diameter ($d_{1}$), PZT thickness ($h_{p}$), epoxy thickness ($h_{e}$), steel membrane diameter ($d_{2}$), and thickness ($h_{m}$). Positive (V${}^{+}$) and ground terminals are indicated. The dashed arrows refer to the inward lateral motion of the PZT. The dashed line refers to the downward bowing motion of the actuator.

The active and passive layers were bonded using conductive epoxy (CircuitWorks CW2400, Chemtronics, Kennesaw, GA, USA). A thin layer of epoxy was applied onto the steel membrane using a 20 μm Jeveka shim-steel stencil. The PZT part was then gently pressed to ensure good contact. The assembled UPM was then cured at 75 °C in a furnace for twenty minutes. The epoxy should be conductive to transfer the PZT bottom terminal to the stainless steel. The steel membrane was then clamped along its circumference and the piezoelectric layer was subjected to an electric field along its poling direction. Due to the inverse piezoelectric effect, the active layer exhibits inward lateral deformation and this causes a bending moment that forces the stainless steel membrane downwards (shown by dashed arrows and dashed line in Figure A1). In this case, the transverse displacement of the steel membrane was amplified by a factor of ≈5 with respect to the calculated free lateral motion of the PZT. Note that the manufacturer’s specifications were used to calculate the free lateral motion [12]. However, there are reports that suggest that piezoelectric properties ($d_{31}$, Elastic modulus) depend on the applied electric field and diverge from the manufacturer’s specifications at high fields [13].

**Figure 2:**
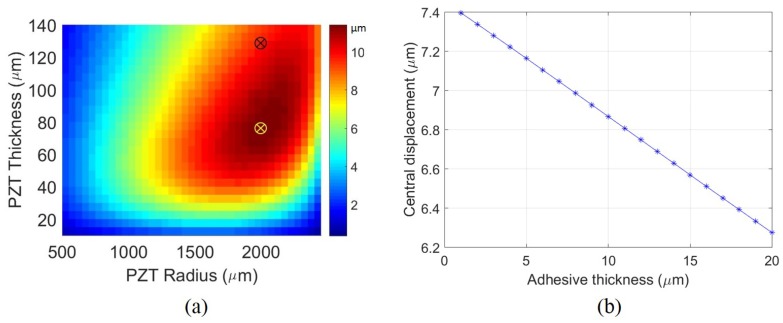
Optimization for maximum actuator displacement: (**a**) Central displacement (shown as color-bar) plotted for different Lead Zirconate Titanate (PZT) parameters using the analytical formulation. The yellow cross is the optimal set of parameters and the black cross is the set of fabricated parameters. Steel diameter = 5 mm, Steel thickness = 50 μm. (**b**) Dependence of microactuator displacement on adhesive thickness using COMSOL Multiphysics v5.3: PZT radius = 2 mm, PZT thickness = 127 μm [12], Voltage = 190 V.

The UPM was optimized to maximize the central displacement for a given voltage to decrease the voltage required to close the valve. An analytical formulation was used from the work done by Mo et al. [14] and is given in Appendix A.1. A constrained optimization study was performed to find the optimal PZT dimensions where the central displacement of the actuator is maximized. This is represented in Figure 2a where the maxima is marked by the yellow cross. As there were no PZT-thinning methods readily available, the final set of parameters was shifted to have higher PZT thickness. This is shown by the black cross. In the optimization study the PZT radius was constrained to not exceed the fixed steel radius of 2.5 mm. The PZT thickness was limited to the plate thickness that was available. The steel thickness was assumed to be fixed at 50 μm as thinner steel layers tended to flex more easily during manual handling. This caused the bonded PZT to crack and fail. The adhesive layer was not considered in the analytical formulation.

Based on prior literature, it was found that the conductive epoxy thickness must be in the order of a few microns to prevent a high displacement loss but also maintain conductivity [15]. As the epoxy was applied manually, the thickness was measured to be ≈15 μm. The effect of epoxy thickness on central displacement was determined using COMSOL Multiphysics v5.3 (COMSOL Inc., Stockholm, Sweden) and is shown in Figure 2b. The final design parameters are shown in Table 1. The fabricated UPM is shown in Figure 4a. The circular actuation region is connected to a square pad. The pad acts as the top electrical terminal and the stainless steel is the bottom terminal. Wires were attached using a press-fit (top terminal) and conductive tape (bottom terminal).

### 2.2. Microchannels and Spacer

The microchannel part has two primary roles: it allows interfacing with the macro-world and provides the base for the valving chamber with inlets and outlets. The former means that there are orifices in the microvalve structure that are far enough apart to interface with external microfluidic connections, and the latter means that a flat valve-seat with small (≤500 μm diameter) and accurate chamber orifices is realizable.

A desktop stereolithography (SLA) printer (Envision-TEC GmbH. Micro Plus Hi-Res, Gladbeck, Germany) was used to fabricate the microchannels. An XYZ resolution of 30 μm × 30 μm × 25 μm can be obtained with Envision-TEC’s proprietary methacrylate/acrylate-based resin (HTM140V2M). The spacer was laser-cut to shape using the same laser cutter as used above. As the spacer thickness defines the valving-chamber height, a very thin machinable material was required. For this purpose, Jeveka shim-steel was used and machined using a laser-cutter.

**Figure 3:**
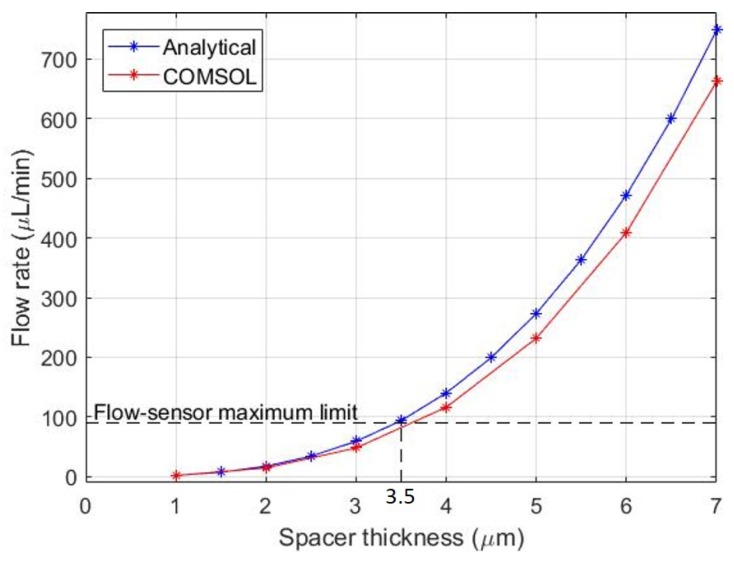
Flow rate dependence on spacer thickness with a 1000 mbar pressure differential.

The microchannels were optimized using the fluid flow rate; de-ionized (DI) water was used as the test fluid. Due to the limited range of the liquid flow sensor that was available, it was desired to have a maximum flow rate of 90 μL min^−1^ at the maximum pressure of 1 bar. The fluid flow was modelled analytically using an electrical-analogue approach [16]. The formulation used is given in Appendix A.2 and the final microchannel dimensions in Table A2. The flow rate was also modelled numerically using COMSOL Multiphysics v5.3 with the Laminar Flow physics module. It was found that the thickness of the spacer was the primary control variable due to the high static resistance of micron-scale passages (shown in Appendix A.2). The flow rate dependence on spacer thickness is shown in Figure 3. It is clear that the analytical and numerical formulations match well. A spacer thickness of 3.5 μm is required so that the maximum flow rate through the microvalve (at 1 bar, fully-open state) is within the range of the available flow-sensor. The closest available spacer was 5 μm in thickness so this was used instead. The hole diameter of the spacer is 4.8 mm.

**Figure 4:**
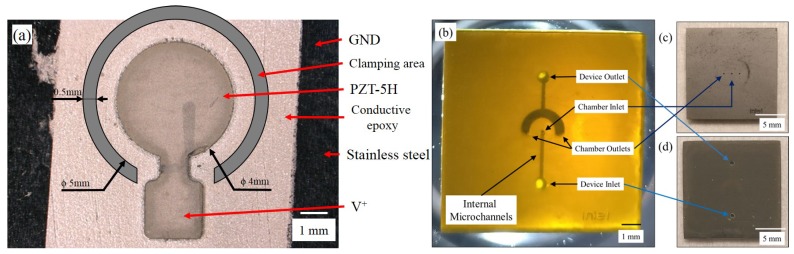
(**a**) Fabricated microactuator with a laser-cut PZT part bonded to a stainless steel membrane using conductive epoxy. The region where the actuator is clamped by the holder is shown in grey. (**b**) 3D printed buried microchannels with inlet and outlet connections. The channels become visible when illuminated with a bright light source underneath. (**c**) Top view of microchannels (**d**) Bottom view of microchannels.

**Figure 5:**
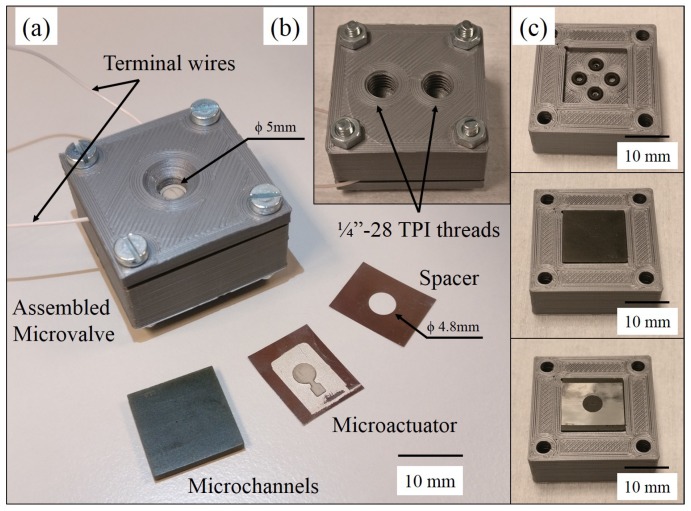
(**a**) Assembled microvalve holder with constituent parts. Terminal wires are copper with 0.5 mm diameter. (**b**) Bottom view of the holder (**c**) First step: O-rings placed in O-ring grooves, second step: microchannels placed in groove, third step: spacer placed over microchannels, fourth step (not shown): actuator is placed and then assembly is clamped.

All critical design parameters are shown in Table 1. The fabricated microchannels are shown in Figure 4 and the spacer is shown in Figure 5a.

### 2.3. Test-Setup

The entire assembly shown in Figure 1b needs to be clamped for the actuator to function and for the assembly to be leak-tight. A valve holder that can clamp the entire microvalve and enable fluidic and electrical interfacing was designed and is shown in Figure 5. A circular clamping surface with 5 mm internal diameter was used to clamp the microvalve assembly using four bolts.

**Figure 6:**
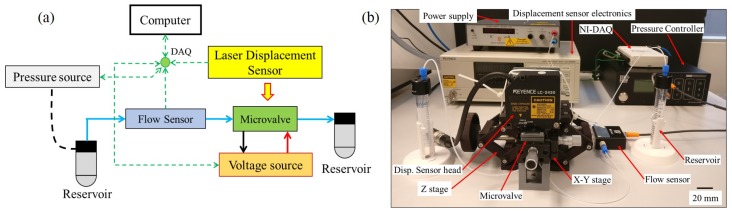
(**a**) Schematic of the test set-up for measuring flow rate and displacement of the microvalve: Green dashed arrows are signal wires, red and black arrows are terminal wires of the microvalve, blue arrows are microfluidic tubing, and the black dashed line is tubing from the pressure source. Double-sided green arrows indicate information transmitted both ways. (**b**) Photograph of the test-setup.

The main characterization of the microvalve is done using the experimental setup shown in Figure 6. The microvalve is connected to a microfluidic circuit consisting of a pressure source (Elveflow OB1 Mk3+, Paris, France) which can apply pressure from −1 bar to 6 bar with respect to ambient, and a thermal flow-sensor (Elveflow MFS3, Paris, France) which can measure a maximum flow rate of 90 μL min^−1^ (accuracy: 5% measured value).

The UPM was controlled by a high-voltage, current-limited, programmable DC power-supply (Delta Elektronika ES0300-0.45, Zierikzee, The Netherlands). A voltage resolution of 0.5 V can be obtained using the programmable interface and a maximum voltage of 300 V can be applied. The current was limited to 1 mA for user safety. The central displacement of the UPM was measured using a high-resolution laser displacement sensor (Keyence LC-2420 sensor-head with a Keyence LC-2400W laser displacement meter, Keyence Corporation, Osaka, Japan). The sensor head has a resolution of 0.01 μm and a working range of ±200 μm. The sensor-head was mounted on a stiff Z-stage for easy adjustment. Command signals to the voltage-source and measurement signals from the displacement sensor were routed through a data-acquisition device (National Instruments NI-DAQ USB-6211, Austin, TX, USA) to a computer. The control program was implemented in Python (2.7).

## 3. Results

The results of the experiments conducted detail the actuation behaviour of the UPM and the valving behaviour of the assembled microvalve.

### 3.1. Actuation Behaviour

The dimensions given in Table 1 were used to manufacture the microactuator shown in Figure 4. The UPM was clamped in a holder and a voltage was applied. The voltage was limited to 150 V for actuator safety. Electrical short circuits were encountered in ≈10% of fabricated actuators at higher voltages (150–190 V) due to a thick epoxy layer (>20 μm) creeping up the side-walls of the PZT prior to epoxy curing.

**Figure 7:**
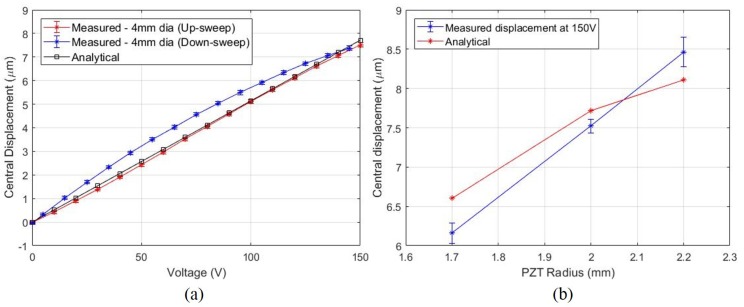
(**a**) Comparison of measured and predicted displacement for a 4 mm diameter actuator in the unimorph piezoelectric microactuator (UPM) holder. (**b**) Displacement of different actuators at 150 V. The predicted displacements are plotted for comparison. Error bars indicate one standard deviation of three measurements in each sample for both graphs.

The measured UPM displacement is plotted alongside the predicted displacements in Figure 7a. The analytical results match closely with the measured displacement, but a characteristic piezoelectric hysteresis was observed. UPMs with different PZT diameters were tested to investigate how the diameter affects displacement. All UPMs were clamped using valve holders with the same internal clamping diameter of 5 mm. The measured central displacement at 150 V has been plotted in Figure 7b with the predicted analytical values.

### 3.2. Valving Behaviour

**Figure 8:**
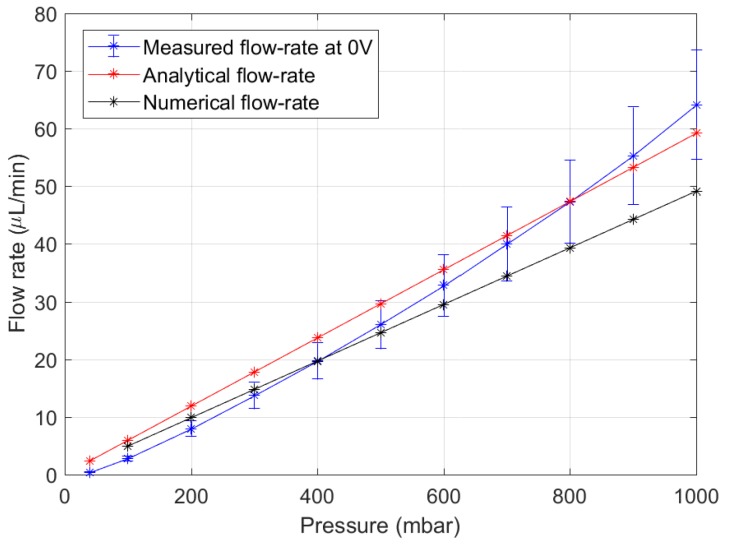
Comparison of predicted and measured flow rate in the microvalve assuming a valving chamber height of 3 μm. Error bars indicate one standard deviation of three measurements.

To verify the validity of the analytical and numerical flow models explained in Section 2.2 and Appendix A.2, increasing pressures were applied across the microvalve and the fluid flow rate was measured. DI water was used as the test fluid. The measured and predicted values are compared in Figure 8. Both the models match well with the analytical results at lower pressures, but the observed flow rate begins to diverge after 500 mbar.

**Figure 9:**
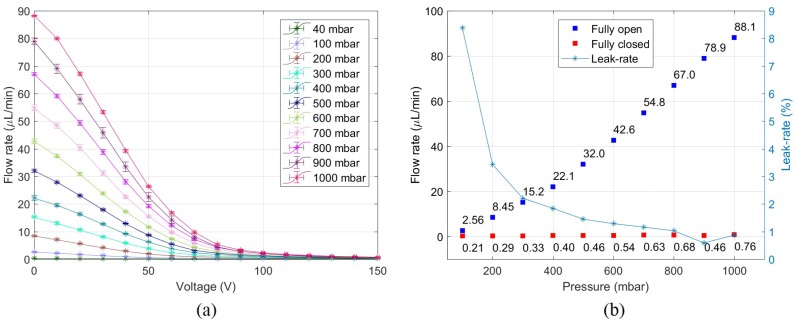
(**a**) Proportional control of flow rate at different pressures. Error bars indicate one standard-deviation of three measurements. (**b**) On-off behaviour and leak-rate of the microvalve at different pressures. Leak-rate is the ratio of closed and open flow rates.

**Figure A3:**
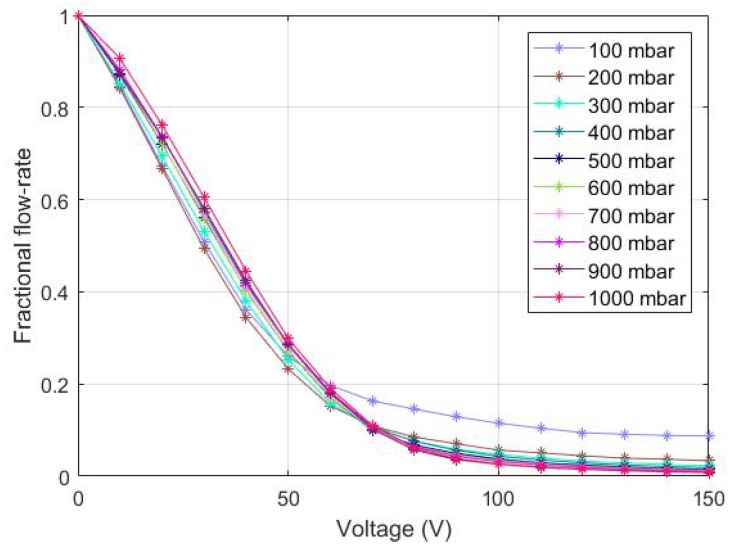
Fractional reduction in flow-rate at different pressures.

The main objective of this work was to prove proportional control of fluid flow. This is shown in Figure 9a. For a set pressure, the voltage was increased from 0 V to 150 V and the flow rate was measured. Increasing the voltage caused the UPM to block the inlet and decrease the flow rate. The same behaviour was observed as pressure was increased with an upward shift in flow rate. The flow rate at fully-open and fully-closed positions at different pressures are shown in Figure 9b. The overlaid leak-rate graph is the ratio of the flow rate at closed and open conditions. To ensure that the valving behaviour was independent of pressure, the fractional reduction in flow for a given voltage can be plotted. This is the ratio of flow rate and the fully-open flow rate at a certain pressure plotted for different voltages. This is shown in Figure A3.

**Figure 10:**
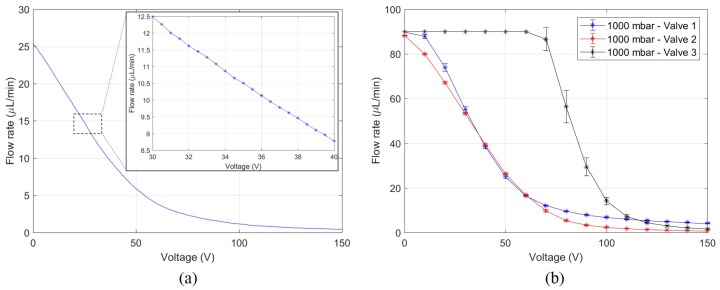
(**a**) Fine control of the microvalve at 500 mbar pressure. (**b**) Reproducibility of flow rate behaviour at 1000 mbar with three microvalves. The flow sensor has a limit of 90 μL min^−1^.

Fine control of the flow rate is shown in Figure 10a. An approximately linear region was observed from 0–50 V. Here, a flow rate change of 0.2 μL min^−1^ was measured for a voltage increment of 0.5 V. The flow rate change was approximately proportional to the inlet pressure. For instance, at higher pressure the flow rate change was higher for a certain voltage increment.

The reproducibility of the microvalve is shown in Figure 10b. Three microvalves with different UPMs (with the same diameter of 4 mm) and microchannels were assembled in three different microvalve holders and their flow rate behaviour was observed. Here, a single flow rate curve at 1000 mbar is shown for each valve. Valves 1 and 2 displayed similar behaviour but Valve 3 showed a much higher flow rate at lower voltage (flow rate saturates at 90 μL min^−1^ due to flow sensor limitations). The main difference between these valves was the clamping pressure applied by the valve holder. A higher pressure was applied in Valves 1 and 2 due to higher torque on the bolts. This was done to push the spacer into the relatively soft microchannel material so that the overall valve chamber height would decrease. The aim of this was to limit the flow rate through the valve to a value that was measurable by the available flow sensor even at 1 bar pressure. The bolts in Valve 3 were not tightened excessively, leading to the change in behaviour seen in Figure 10b.

## 4. Discussion

### 4.1. Actuation

In Figure 7a, it is clear that the up-sweep path is different from the down-sweep path of displacement behaviour. Measurements were made after 20–30 cycles of actuation due to which poling effects can be eliminated as the source of this difference in behaviour. This means that piezoelectric hysteresis is the main reason. Hysteresis in piezoelectric actuators is one of the major causes for positioning inaccuracies [17]. Hysteresis models exist that could be used to relate the voltage to displacement, but it is beyond the scope of this work.

The displacement behaviour for UPMs with different PZT diameters was tested and has been shown in Figure 7b. The error bars indicate the standard deviation of three cycles of actuation (One cycle = 0 V → 150 V → 0 V) of each UPM. The measured results show a slightly different trend than that predicted. It appears that deflection is increasing at a constant rate in the measurements, but the analytical values show a clear decrease in slope as the radius increases. Actuators with higher radii PZT need to be tested to find where the slope changes. A maximum central displacement of 8.5 μm at 150 V was measured using the 4.4 mm dia UPM.

To test repeatability of the UPM fabrication procedure, three UPMs (PZT dia—4 mm) were fabricated in the same batch and subjected to 150 V. The central displacement measured was 7.41±0.36 μm. The primary difference between the actuators was the adhesive thickness, which was measured to be 12.6±3.7 μm. Automating the process of applying epoxy might be a good solution to improving repeatability.

### 4.2. Valving

Figure 8 shows that the microvalve behaviour can be predicted with good accuracy using both the analytical and numerical models. This, of course, is dependent on our assumption that the spacing of the microvalve has decreased to 3 μm due to tightening of the holder bolts. This was verified with reasonable confidence by observing the displacement of the UPM without any fluidic pressure. The UPM did not move downwards after 3 μm meaning that it contacted the valve seat. This is the valving chamber height.

It is seen from Figure 8 that the measured flow rate matches accurately with the numerical model at lower pressures, but diverges at 500 mbar. This is because the roof of the valve-chamber, which is the UPM, deforms outward at these pressures, allowing a higher flow rate to pass. The numerical model did not account for this expansion as the computing time was too high. The analytical formulation predicts a higher flow rate so it is a good conservative metric to use when designing future microvalves. Including fluidic capacitance in the analytical expression will allow the trends of the two curves to match.

Proportional control of flow rate in the microvalve is clearly observed in Figure 9a. A uniform upward shift in the flow rate curve is seen in relation to the pressure differential. The small standard deviation (error bars) shows that there is good repeatability of the valving behaviour. It can also be seen that, at all pressures, a bulk of the valving is done from 0–100 V. The slope changes rapidly from 90 V and the flow rate appears to asymptotically decrease to zero. In the 1000 mbar curve, flow rate decreases at an average rate of 0.95 μL min^−1^ V^−1^ till 100 V, but this changes to 0.04 μL min^−1^ V^−1^ in the latter half of the curve. It is hypothesized that the UPM membrane contacts the valve-seat at 100 V, but due to surface defects, a large leakage flow is still observed. Increasing the voltage further causes the membrane to flatten itself against the valve seat and close the chamber orifice more efficiently.

The leakage behaviour of the microvalve is shown in Figure 9b. Here, leakage rate is defined as the ratio of closed and open flow rates. Generally, leakage is measured using helium gas but this facility was not available [10]. A high leakage-rate is observed at low pressures; this is because flow rate at open condition is low, but a constant leakage is always present due to valve seat defects. As pressure increases, open flow rate increases while closed flow rate remains relatively constant, effectively decreasing the leakage-rate. Some methods to decrease leakage include decreasing the valve-seat area by using a knife-edge contact and introducing a soft material like PDMS or a parylene layer to the actuator or valve-seat.

To investigate fine control of the flow, voltage increments of 0.5 V were applied to the microvalve at 500 mbar pressure. This was the voltage resolution of the power supply. The resulting curve is shown in Figure 10a. In the linear regime, a flow rate change of 0.2 μL min^−1^ was measured. This is dependent on the pressure differential across the microvalve. Higher flow rate changes were measured at higher pressures and vice versa.

An important characteristic of any device is its reproducibility. The flow rate behaviour of three different microvalves are shown in Figure 10b. The dimensions of the microvalves were similar, but there is a clear difference in their behaviour. Although valves 1 and 2 show similar behaviour, valve 3 acts very differently. This is because lower torque was applied while tightening the valve holder bolts in valve 3. Decreasing the torque results in a lower clamping force on the microvalve. This means the valve chamber height is larger than valves 1 and 2. This allows more fluid to pass through the microvalve. Valve 1 and 2 have bolts that were tightened with higher torque, resulting in higher clamping force, leading to a lower valve chamber height and a lower flow rate. This was done so that the flow rate could be measured using the available flow sensor over the entire pressure range of the experiment. The decrease in valve-chamber height is due to the spacer being pushed into the microchannel material, thereby permanently deforming it. Lower clamping force is therefore preferred. The reproducibility is then linked closely to the clamping force applied to the microvalve. The valve chamber height was measured using the maximum UPM displacement at 150 V. The UPMs of valves 1 and 2 deformed by 2.9 μm and 2.8 μm respectively while valve 3 deformed by 5.1 μm.

As this is a normally-open microvalve, for applications that require long periods of closed state it is essential that the power consumption be minimal. To obtain proportional behaviour, the microvalve is operated in quasi-static mode with the external power supply consuming 37.5 μW. Similar piezoelectric microvalves have shown a static power consumption of 2500 μW [18], and 3000 μW [10]. At least to the authors’ knowledge, no piezoelectric microvalve was found that consumes such low static power in this pressure range.

## 5. Conclusions

A proportionally-controlled piezoelectric microvalve was designed, fabricated and characterized. DI water at room temperature was used for characterization. The uniqueness of this work is that a novel combination of rapid-prototyping methods like stereolithography for three-dimensional microchannels and laser-cutting for spacer and actuator fabrication were used. Using shim-steel as an accurate and easily modifiable spacing method for such a device has also been demonstrated for the first time. The fabrication methodology detailed above is cheaper than silicon Microelectromechanical System (MEMS) devices and is suitable for small batch production. These techniques can be extended to design other microfluidic devices like micropumps and flow sensors.

The specifications of the final microvalve are given in Table 2. A thorough characterization of the unimorph microactuator was performed and a good match was found between the analytical and measured results. A high central displacement of 7.5 μm was observed for an actuator dimension of 5 mm × 5 mm × 0.2 mm (PZT diameter = 4 mm). Due to limitations in the available flow sensor, the full potential of the microvalve could not be explored. The projected flow rate range for this microvalve is 0–750 μL min^−1^ (water). A low power consumption of 37.5 μW was measured.

## Figures and Tables

**Table 1:** Microvalve design parameters.

Parameter	Analytical	Numerical	Fabricated
PZT diameter	4 mm	4.2 mm	4 mm
PZT thickness	76 μm	68 μm	127 μm
Epoxy thickness	-	<5 μm	≈15 μm
Steel diameter	5 mm	5 mm	5 mm
Steel thickness	25 μm	25 μm	50 μm
Spacer thickness	3.5 μm	3.6 μm	5 μm

**Table 2:** Obtained Microvalve specifications.

Specification	Measured Value
Flow range	0–90 μL min^−1^
Flow control resolution	0.2 μL min^−1^ at 500 mbar
Leakage	0.8% open-flow at 1000 mbar
Max. differential pressure	1 bar
Static power consumption	37.5 μW
Operating voltage	0–150 V
Dimensions (effective)	5 mm × 5 mm × 1.8 mm

**Figure A2:**
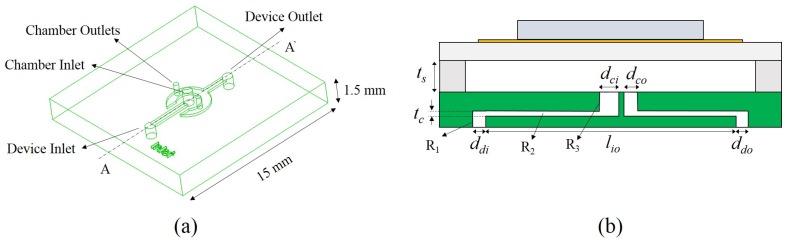
(**a**) Transparent view of the microchannels: Internal channels are visible. The microchannel part is large (15 mm) to enable easy manual handling. (**b**) Cross-section of microchannels along A-A’ with spacer and microactuator: Design parameters are spacer thickness ($t_{s}$), device inlet diameter ($d_{di}$), device outlet diameter ($d_{do}$), distance between device orifices ($l_{io}$), chamber inlet diameter ($d_{ci}$), chamber outlet diameter ($d_{co}$), internal channel thickness ($t_{c}$). Static resistances are represented as $R_{i}$.

## Abbreviations

The following abbreviations are used in this manuscript:

UPM	Unimorph Piezoelectric Microactuator
MEMS	Microelectromechanical System
PZT	Lead Zirconate Titanate

## Appendix A

### Appendix A.1. Optimization of Microactuator Parameters

The deflection of a UPM where the two layers have unequal diameters ($d_{1}<d_{2}$ from Figure A1) was derived by Mo et al. [14]. Equation (A1) shows the relationship between the downward displacement of the membrane (*w*), and the parameters of the unimorph (Figure A1) [14]. These parameters are given in Table A1. Note that a lateral dimensional constraint of 5 mm × 5 mm was assumed to provide a starting point. The primary control variables are the PZT layer dimensions.

**Table A1:** Microactuator parameters.

Material	Parameter	Symbol	Value
PZT	Young’s Modulus	$E_{11}$	62 GPa
	Compliance constant	$S_{11}^{E}$	$1/E_{11}$
	Piezoelectric constant	$d_{31}$	$-320\times 10^{-12}$ mV^−1^
	Poisson’s ratio	ν	0.31
	Thickness	$h_{p}$	**To be optimized**
	Radius	$r_{1}=d_{1}/2$	**To be optimized**
	Polarization field	$E_{p}$	$1.5\times 10^{6}$ V m^−1^
	Voltage	V	$0\leq V\leq Ep\times h_{p}$
Steel	Young’s Modulus	$E_{m}$	193 GPa
	Compliance constant	$S_{m}$	$1/E_{m}$
	Thickness	$h_{m}$	≥50 μm
	Radius	$r_{2}=d_{2}/2$	≤2.5 mm

The maximum voltage that can be applied to the UPM is given in the table above. This is the potential that was applied during poling of the material by the manufacturer. Increasing the voltage beyond this can degrade PZT behaviour.

(A1) $$w\left(r\right)=\left\{\begin{array}{c} \frac{C_{1}\left\{2r_{1}^{2}\ln\left(\frac{r_{1}}{r_{2}}\right)+\left[1-{\left(\frac{r_{1}}{r_{2}}\right)}^{2}\right]r^{2}\right\}V}{C_{2}-C_{3}{\left(\frac{r_{1}}{r_{2}}\right)}^{2}+\frac{1}{2}h_{p}^{4}S_{m}^{2}\left(1+v\right){\left(\frac{r_{1}}{r_{2}}\right)}^{4}}\\ r⩽r_{1}\\ \frac{C_{1}\left\{2r_{1}^{2}\ln\left(r\right)-r_{1}^{2}\left[2\ln\left(r_{2}\right)-1\right]-{\left(\frac{r_{1}}{r_{2}}\right)}^{2}r^{2}\right\}V}{C_{2}-C_{3}{\left(\frac{r_{1}}{r_{2}}\right)}^{2}+\frac{1}{2}h_{p}^{4}S_{m}^{2}\left(1+v\right){\left(\frac{r_{1}}{r_{2}}\right)}^{4}}\\ r_{1}<r⩽r_{2} \end{array}\right.$$

where

(A2) $$\begin{array}{c} C_{1}=3d_{31}h_{m}S_{11}^{E}S_{m}\left(h_{m}+h_{p}\right)\\ C_{2}=4S_{11}^{E}h_{p}h_{m}^{3}S_{m}+6S_{11}^{E}h_{m}^{2}h_{p}^{2}S_{m}+4S_{11}^{E}h_{m}h_{p}^{3}S_{m}\\ \;+\frac{1}{2}h_{p}^{4}S_{m}^{2}\left(1+v\right)+\frac{2h_{m}^{4}S_{11}^{E^{2}}}{1+v}\\ C_{3}=4S_{11}^{E}h_{p}h_{m}^{3}S_{m}+6S_{11}^{E}h_{m}^{2}h_{p}^{2}S_{m}+4S_{11}^{E}h_{m}h_{p}^{3}S_{m}\\ \;+h_{p}^{4}S_{m}^{2}\left(1+v\right) \end{array}$$

where w(r) is the transverse displacement of the membrane along the radius *r*.

### Appendix A.2. Optimization of Microchannel Parameters

An electrical analogy is best suited for calculating flow-rates in microfluidic circuits. In such an analogy, the pressure differential is similar to potential difference, fluid flow is similar to current, and the drag or pressure drop due to the wetted path is similar to electrical resistance. Also, high pressures can deform channels and cause flow-rate to increase. This is modelled as a capacitance. In this case, capacitance is neglected due to its low influence.

All the channels were modelled as static resistances and the valving chamber was modelled as a variable resistance [16]. Rectangular and circular channel resistances are shown in Equations (A3) and (A4) respectively. Figure A2 shows detailed representations of the microchannels to be optimized.

(A3) $$\begin{array}{cc} \; & R_{sr}=\frac{12l\mu }{wh^{3}\left(1-0.63\frac{h}{w}\right)} \end{array}$$

(A4) $$\begin{array}{cc} \; & R_{sc}=\frac{8\mu l}{\pi r^{4}} \end{array}$$

where μ is the dynamic viscosity, *l* is channel length, *w* is rectangular channel width, *h* is channel height, and *r* is circular channel radius.

The variable chamber resistance is:

(A5) $$R_{var}=\frac{6\mu \ln\frac{a_{2}}{a_{1}}}{\pi s^{3}}$$

where $a_{1}$ is chamber inlet radius, $a_{2}$ is valve seat radius ($\approx 5a_{1}$), and *s* is the chamber height (=spacer thickness, $t_{s}$).

Based on the channel configuration, channel resistances can be added up to obtain the total static resistance $R_{s}$. The flow-rate *Q* is then calculated as:

(A6) $$Q=\frac{\Delta P}{R_{s}+R_{var}}$$

where ΔP is the pressure differential.

The design parameters are tabulated in Table A2. The dimensions $d_{ci}$, $d_{co}$, and $t_{c}$ were empirically found to be the smallest printable dimensions with the SLA printer available.

The static resistances of the microchannels are tabulated in Table A3. The resistance indices are shown in Figure A2b. $R_{chamber}$ refers to the static resistance of the chamber with a 5 μm height. The chamber height is determined by the spacer thickness.

**Table A2:** Microchannel and Spacer Design Parameters.

Parameter	Value
Chamber inlet dia ($d_{ci}$)	0.2 mm
Chamber outlet dia ($d_{co}$)	0.2 mm
Internal Channel thickness ($t_{c}$)	0.2 mm
Distance b/w device orifices ($l_{io}$)	8 mm
Device orifice dia ($d_{di}$, $d_{do}$)	0.8 mm
Spacer thickness ($t_{s}$)	5 μm

**Table A3:** Microfluidic resistances.

Resistance	Value [Pas/m^3^]
$R_{1}$	$1.7\times 10^{8}$
$R_{2}$	$4.81\times 10^{4}$
$R_{3}$	$4.5\times 10^{10}$
$R_{chamber}$	$2.2\times 10^{13}$

It is evident from Table A3 that the valve chamber resistance is much higher than any other part of the circuit.

### Appendix A.3. Microvalve Behaviour

To ensure that the valving behaviour of the microvalve is independent of the pressure, the fractional flow rate can be observed. This is the ratio of the flow rate and fully-open flow rate at different voltages. The behaviour at different pressures is shown in Figure A3. The behaviour appears to be similar at all pressures, meaning the functioning of the valve is independent of the pressure.
